# Prevalence of head lice infestation and pediculicidal effect of permethrine shampoo in primary school girls in a low-income area in southeast of Iran

**DOI:** 10.1186/s12895-017-0062-9

**Published:** 2017-07-24

**Authors:** Moussa Soleimani-Ahmadi, Seyed Aghil Jaberhashemi, Mehdi Zare, Alireza Sanei-Dehkordi

**Affiliations:** 1 Social Determinants in Health Promotion Research Center, Hormozgan University of Medical Sciences, Bandar Abbas, Iran; 2 Department of Medical Entomology and Vector Control, Faculty of Health, Hormozgan University of Medical Sciences, P.O. Box: 79145–3838, Bandar Abbas, Iran; 3 Bashagard Health Center, Hormozgan University of Medical Sciences, Bashagard, Iran; 4 Department of Occupational Health Engineering, Faculty of Health, Hormozgan University of Medical Sciences, Bandar Abbas, Iran

**Keywords:** Head lice infestation, Schoolchildren, Socio-demographic characteristics, Permethrin shampoo, Bashagard, Iran

## Abstract

**Background:**

Head lice infestation is a common public health problem that is most prevalent in primary school children throughout the world, especially in developing countries including different parts of Iran. This study aimed to determine the prevalence and risk factors associated with head lice infestation and pediculicidal effect of 1% permethrin shampoo in primary schools girls of Bashagard County, one of the low socioeconomic areas in southeast of Iran.

**Methods:**

In this interventional study six villages with similar demographical situations were selected and randomly assigned into intervention and control areas. In each area 150 girl students aged 7–12 years were selected randomly and screened for head lice infestation by visual scalp examination. In intervention area, treatment efficacy of 1% permethrin shampoo was evaluated via re-examination for infestation after one, two, and three weeks. Pre-tested structured questionnaire was used to collect data on socio-demographic and associated factors of head lice infestation.

**Results:**

The prevalence of head lice infestation was 67.3%. There was significant association between head lice infestation and school grade, family size, parents’ literacy, bathing facilities, frequency of hair washing, and use of shared articles (*p* < 0.05). The effectiveness of 1% permethrin shampoo for head lice treatment was 29.2, 68.9, and 90.3% after the first, second, and third weeks, respectively.

**Conclusion:**

The head lice infestation is a health problem in primary school girls of Bashagard County. Improvement of socioeconomic status and providing appropriate educational programs about head lice risk factors and prevention can be effective for reduction of infestation in this area.

**Trial registration:**

This trial has been registered and approved by Hormozgan University of Medical Sciences ethical committee (Trial No.764). Trial registration date: March 17 2014.

## Background

The human head louse, *Pediculus humanus capitis*, is an obligate ectoparasitic insect, which is readily transmitted by direct head-to-head contact, especially in crowded conditions [1]. Head lice infestation is a common health problem in children worldwide and a survey conducted in the south of Iran reported the prevalence to be 23.9% [2].

Although the biology of head lice is the same globally, the epidemiology depends on the society and cultural behaviour. Treatment options are dependent on the context and factors such as access to pediculicides and availability of educational interventions for adoption of a particular behaviours [3, 4]. For the treatment of head lice infestation two classes of insecticides are commonly used, organophosphates such as malathion and pyrethroids such as permethrin and phenothrin with pyrethroids being the most widely used ones due to their shorter contact time and less odor [5]. This is despite the fact that the efficacy of many of the insecticide products including permethrin has been now reduced because the head louse has acquired resistance to some of these chemicals [6, 7].

This study was conducted aiming to determine the prevalence and risk factors associated with head lice infestation and treatment efficacy of 1% permethrin shampoo in primary schools girls of Bashagard County, one of the low socioeconomic areas in the southeast of Iran.

## Methods

### Study design and data collection

This interventional community-based cross-sectional study was carried out in Bashagard County in the Hormozgan province, southeast of Iran. The county has an area of 16,000 km^2^ and is located between latitudes 26°04′-26°58′ N and longitudes 57°23′-59°02′ E with an approximately 43,000 population in 2016.

**Fig. 1:**
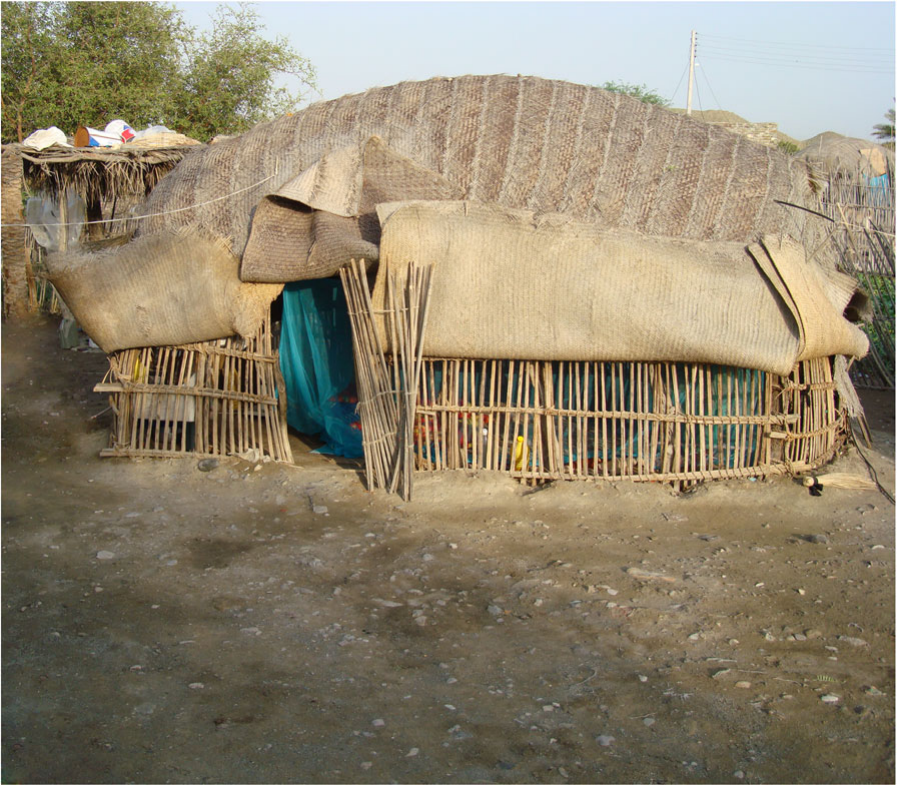
Sheds made of palm leaves, a living place for people in Bashagard County, southeast of Iran

Bashagard County has a warm climate with mean annual temperature of 27.8 °C ranging from 18.8 to 38 °C. The rainfall occurs through the January–October with a total annual average of 235.9 mm during 2015–2016. The annual averages of minimum and maximum relative humidity are respectively 16% in June and 38.2% in August. It is a low socioeconomic area with majority of the population living in houses made of cement and blocks and shelters made of palm tree branches (Fig. 1).

**Fig. 2:**
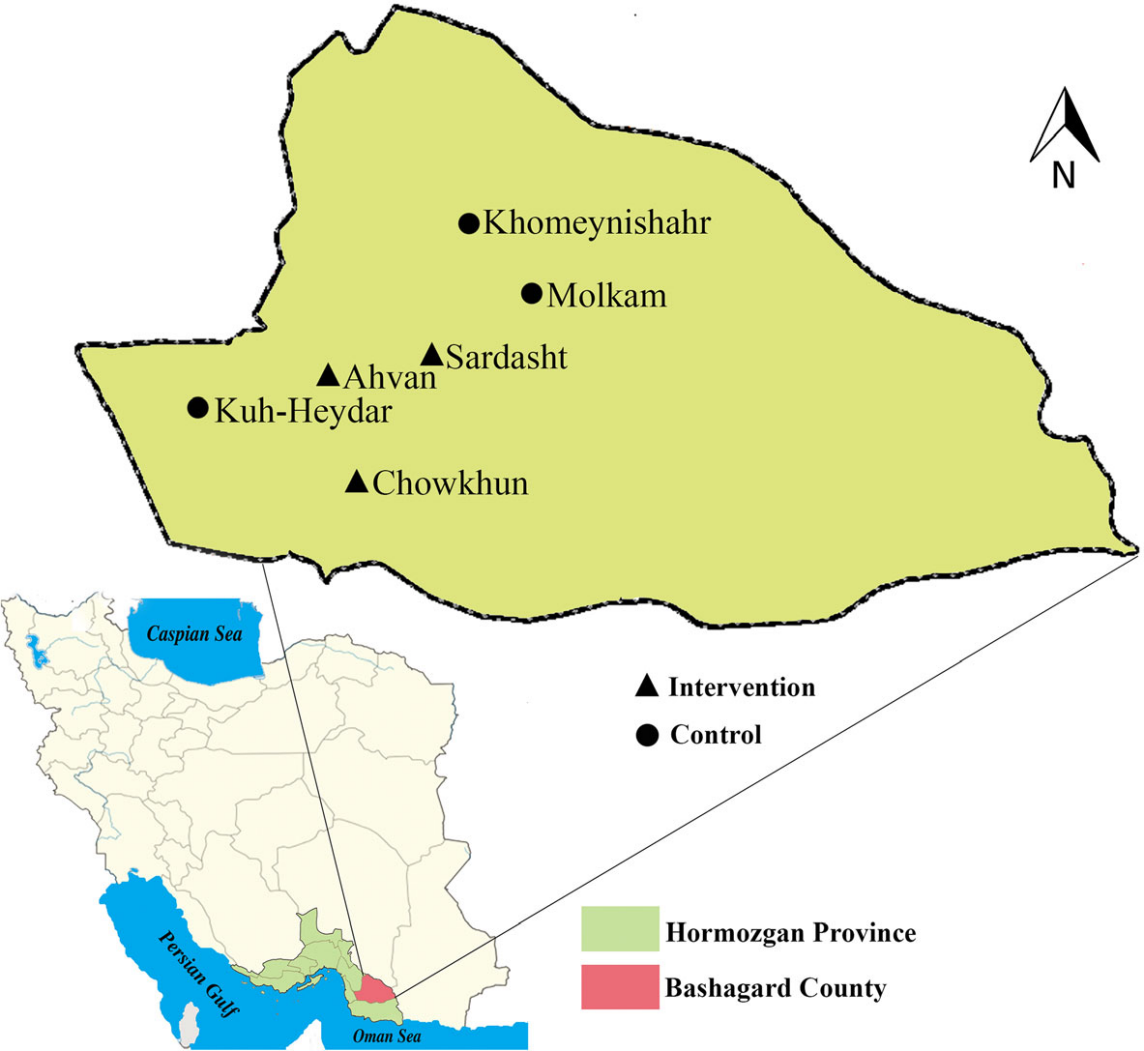
Map showing the provinces of Iran, highlighting the location of Hormozgan province and study villages in Bashagard County, southeast Iran

On the basis of available epidemiological data and unpublished data on the prevalence of head lice infestation in the Bashagard health center, six villages with similar topographical, epidemiological, and demographic characteristics were selected for conducting the study and assigned randomly into intervention and control areas which received and did not received 1% permethrin shampoo, respectively. In each area 150 girl students aged 7–12 years were selected randomly. The study area is shown in Fig. 2.

A team including health workers of the study area and a medical entomologist inspected the hair and scalps of the students visually for eggs, nymphs, and adult lice.

The entire head was examined carefully and special attention was paid to the nape of the head and behind the ears, for a period of 5 min. Students whose hair had at least one of the developing stages of louse including egg, nymph, and adult was considered as head lice infested. In this regard the nits found less than about 1/4″ away from the scalp and eggs with a dark colour considered as viable. After the examination, each student was interviewed using a pre-tested structured questionnaire. The questionnaires were administered by trained field interviewers and supervised by the principal investigator. The questions included respondents’ demographic characteristics, family size, parent’s educational level and job, history of head lice infestation in family members, bathroom availability in the home, bathroom, and dwelling houses construction materials, electricity, and water supply.

In the intervention area, each student directly received three 60 ml bottles of 1% permethrin shampoo and through a face to face educational program students were advised to use shampoo at 7 day intervals and each time the shampoo should left on head for 10 min. The effectiveness of shampoo was evaluated via reexamination for head lice infestation after one, two, and three weeks and the therapeutic efficacy was calculated as the number of cured divided by the total number of initial infected cases.

Students were considered as infestation free if they did not have any of the developing stages of louse including egg, nymph, and adult. The therapeutic efficacy of permethrine shampoo was calculated as the number of cured cases divided by the total number of infested cases.

### Statistical analysis

The data were analyzed using SPSS version 19. Descriptive statistics were used to show percentages, averages, and relative frequencies of the variables. Cross tabulation of variables and Chi-squared test were used to determine the statistical significance of differences of relative frequencies. The results were considered significant at 5% levels of significance (*p* < 0.05).

### Ethical consideration

Students of study villages and their family were informed about the objectives and procedures of the investigation. The parents signed a consent form and the students were informed that their participation was purely voluntary and they were free to withdraw from the study at any time. In this study identification numbers were used instead of participant names and collected data were kept confidential.

## Results

A total of 300 female students were interviewed. The ages of students ranged from 7 to 12 years with an average of 9.45 ± 1.15 years. The mean family size was 5.3 ± 2.1 people ranging from 2 to 12 people.

During this study, a total of 202 (67.3%) students were found to be infested with at least a single specimen of louse. Infestation rate was estimated 68.6% and 66% in the intervention and control groups, respectively. In 74 (36.6%) of the positive students, only louse eggs were found, whereas in 128 (63.4%) students, at least one of the live adult, nymph, and viable nit was observed.

The prevalence of head lice infestation was significantly higher in the highest grade students who aged 9 years (*p* < 0.012), and the lowest infestation rate was in 7 years age grade (Table 1).

**Table 1:** Head lice infestation in female primary school girls by socio- demographic characteristics in Bashagard County, southeast Iran

Characteristics	Examinations (n)	infestations (%)	*p*-value^a^
School grade(age)			0.012
I(7)	50	50	
II(8)	52	57.7	
III(9)	43	81.4	
IV(10)	51	76.4	
V(11)	57	71.9	
VI(12)	47	68.1	
Bathroom within the house			0.038
Yes	186	60.7	
No	114	78.1	
Frequency of hair washing per week			0.021
1	211	72.9	
2	78	56.4	
≥ 3	11	27.2	
Sharing articles^b^			0.043
Yes	170	71.7	
No	130	60	

^a^Chi-square test

^b^combs, and scarves

In this study, the frequency of lice infestation was significantly higher (78.1%) among schoolchildren with no bathroom in their homes compared to those with bathroom in their homes (60.7%) (*p* < 0.038). Moreover, frequency of hair washing had significant relationship with infestation rate (*p* < 0.021). Students who regularly washed their hair three times or more per week had the least head lice infestation compared with the students who washed hairs once a week (Table 1).

As Table 1 indicates, the frequency of infestation is significantly higher among students who used shared articles such as combs and scarves (71.7%) compared with students who did not use shared articles (60%) (*p* = 0.043).

The study results also showed that head lice infestation rate was significantly related to the mothers’ (*p* = 0.032) and fathers’(*p* = 0.021) educational levels and it was 82.4% and 81% in students with uneducated father and mother, respectively. Moreover, the students whose father and mother’ educational level was higher than diploma had 6.7 and 14.3% infestation, respectively. Parents’ job was not significantly associated with lice infestation (Table 2).

**Table 2:** Head lice infestation in primary school girls according to family demographic characteristics in Bashagard County, southeast Iran

Characteristics	Examinations (n)	Infestations (%)	*p*-value^a^
Father’s education			0.021
Illiterate	159	82.4	
Primary	109	58.7	
Secondary	17	35.3	
High school / University	15	6.7	
Mother’s education			0.032
Illiterate	137	81	
Primary	142	57	
Secondary	14	64.3	
High school / University	7	14.3	
Father’s Job			0.89
Employee	259	67.6	
Self-employment	41	65.9	
Mother’s Job			0.31
Employee	14	50	
Housewife	286	68.2	
Family size			0.0001
2–3	7	28.6	
4–5	92	52.1	
6–7	122	73.5	
≥ 8	79	79.7	

^a^Chi-square test

Analysis of another factors influencing head lice infestation showed that infestation rate was positively associated with family size of students (*p* = 0.0001). The prevalence of head lice infestation according to family demographic characteristics is demonstrated in Table 2.

Rates of head lice infestation detected at initial, 7, 14, and 21 days in intervention and control groups are reported in Table 3.

**Table 3:** Head lice infestation rate in intervention and control groups at initial, 7, 14, and 21 days during examinations

Examiation time	Intervention group		Control group		*p*-value^a^
	Examined (n)	Infested n(%)	Examined (n)	Infested n(%)	
Initial	150	103 (68.6)	150	99 (66.0)	0.622
7 days	148	73 (49.3)	149	98 (65.7)	0.003
14 days	150	32 (21.3)	150	96 (64.0)	0.0001
21 days	147	10 (6.8)	148	92 (62.1)	0.0001

^a^Chi-square test

**Fig. 3:**
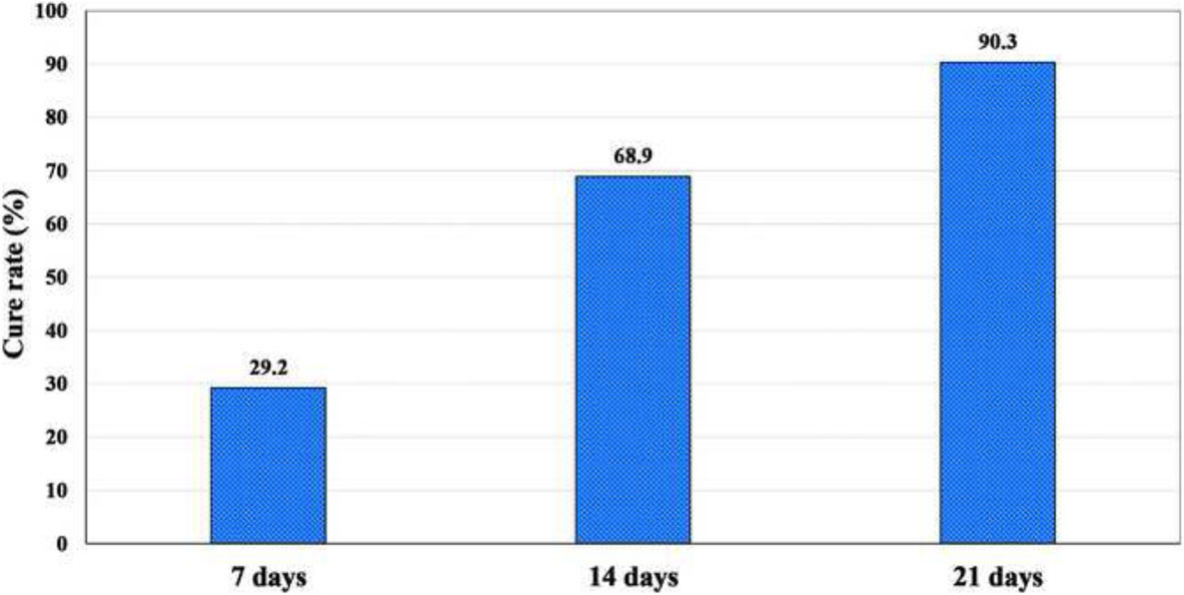
Cure rates after 7,14,and 21 days of treatment with 1% permethrin shampoo in the intervention group in Bashagard County, southeast Iran

In this study, the effectiveness of 1% permethrin shampoo was revealed to be 29.2%, 68.9%, and 90.3% at follow-up examinations after one, two and three weeks, respectively (Fig. 3).

## Discussion

Infestation with head lice is a common health problem that is most prevalent in primary schools throughout the world, especially in developing countries including different regions of Iran [8, 9]. In this study the prevalence of head lice infestation was 67.3% in primary school girls which is higher than the mean of infestation rate which has been reported to be 8.8% in different parts of Iran [9]. High prevalence of head lice infestation in the study area can be attribute to factors such as low parents’ educational level, use of shared personal hygiene items, large family size, poor health facilities, low frequency of bathing per week, lack of a school health educator, and low socioeconomic status. Obviously, many of these factors are due to the extreme poverty.

Results of the studies in primary schoolchildren from different parts of Iran show the infestation rate between 0.47% to 27.1% [9, 10] and the rate of lice infestation among school children in some Middle East and other regional countries ranges from 4.2 to 78% [11].

The variation of infestation rate may be due to several factors including personal hygiene, family size, economic condition and family income [9, 12].

In this study the highest rate of infestation was seen in 9-year-old students. This finding is similar to some studies in different parts of the world [8–10]. This can be explained by behavioral factors which make children at this age to have more direct physical contact with friend. Physical contacts, especially head-to-head contacts are the most important factors in transmission of head lice infestation [13].

In this study, there was a significant association between head lice infestation and the presence of bathroom in the home as well as the frequency of hair washing. This finding is supported by other studies in the northwest of Iran [9, 14]. Similarly, in studies conducted in Korea, Jordan, and Egypt, a strong association between head lice infestation and bathing facilities in the home was reported [15–17].

According to the results, use of shared articles such as combs, hair brushes, and scarves affects the head lice prevalence. Similar findings have been reported from Jordan, Egypt, Palestine, and Yemen [16, 18–20]. All the girls in this study were wearing scarves due to their Islam religion. Covering the head may facilitate the infestation because of creating a better and ideal scalp humidity and temperature for the head lice to thrive and multiply [21].

The results of this study also showed that the infestation was more common among students with low-educated parents. This finding is in agreement with results of previous studies which carried out in Iran, Egypt, Yemen, Palestine, and Turkey [9, 17, 19, 20, 22], which have shown that low educational levels of mothers and fathers increased the risk of infestation. The reason is that educated mothers and fathers have more information about head lice infestation and its prevention due to their awareness and social communication [23, 24]. Since educational intervention has been reported to be effective in increasing community involvement and reduction of prevalent insect-borne diseases in low socioeconomic areas such as Bashagard County [25], it is important to provide appropriate educational programs directed toward parents, teachers, and students to increase community awareness about head lice risk factors and prevention.

This study also showed a positive relationship between head lice infestation and family size.

This finding is in line with the results of other studies in different parts of Iran [16]. Similarly, in studies conducted in Korea, Jordan, Egypt, Yemen, Malaysia, and Turkey head lice infestation prevalence was more common among big size families [15–17, 19–21].

In an overcrowded home, close contact between family members facilitates the transmission of head lice. Moreover, having more children may lead to higher infestation rates because parents pay less time per child to perform laundry and personal cleansing.

In this study, the effectiveness of 1% permethrin shampoo after the first, second, and third weeks were 29.2, 68.9, and 90.3%, respectively. A similar study in the south of Iran reported that 1% permethrin shampoo was effective as pediculicide product in schoolchildren with 64.1% and 89.7% cure rate after 6 and 14 days, respectively [26].

The dominant therapeutic effect of permethrin shampoo in our study can be attributed to effective face to face educational intervention and subsequent follow ups of infested cases.

Since the large insecticide selection pressure induced by conventional insecticides has led to the emergence and spread of resistance in many parts of the world [27, 28] which may lead to treatment failure, there is a need for regular monitoring of insecticide resistance in order to select suitable insecticides for successful control of head lice infestation.

## Conclusion

Our study revealed that there is a high prevalence of head lice infestation among primary school girls in Bashagard County. Factors that may explain the high prevalence of head lice infestation in the studied girls may be attributed to low parents’ educational level, shared use of personal hygiene items, large family size, poor health facilities, low frequency of bathing per week, lack of a school health educator, and low socioeconomic status. Moreover, since in this study 1% permethrin shampoo found to be suitable as a pediculicide for head lice infestation control in schoolchildren, regarding the socioeconomic status of Bashagard county population, free distribution of 1% permethrin shampoo and providing educational program for proper and regular use of the shampoo seems necessary for control of head lice infestation in primary school girls.
